# Validation of the WHO Disability Assessment Schedule (WHODAS 2.0) 12‐item tool against the 36‐item version for measuring functioning and disability associated with pregnancy and history of severe maternal morbidity

**DOI:** 10.1002/ijgo.12465

**Published:** 2018-05-23

**Authors:** Carla Silveira, Renato T. Souza, Maria L. Costa, Mary A. Parpinelli, Rodolfo C. Pacagnella, Elton C. Ferreira, Jussara Mayrink, José P. Guida, Maria H. Sousa, Lale Say, Doris Chou, Veronique Filippi, Maria Barreix, Kelli Barbour, Tabassum Firoz, Peter von Dadelszen, José G. Cecatti, the Brazilian Cohort on Severe Maternal Morbidity (COMMAG) study group and the WHO Maternal Morbidity Working Group (MMWG), Carla B. Andreucci, Carla B. Andreucci, Carina R. Angelini, Carina R. Angelini, Juliana P. Ferraz, Juliana P. Ferraz, Dulce M. Zanardi, Dulce M. Zanardi, Rodrigo S. Camargo, Sara Cottler, Olubukola Fawole, Luis Gadama, Atf Ghérissi, Gill Gyte, Michelle Hindin, Anoma Jayathilaka, Amanda Kalamar, Yacouba Kone, Nenad Kostanjsek, Isabelle Lange, Laura A. Magee, Arvind Mathur, Affette McCaw‐Binns, Mark Morgan, Stephen Munjanja, Gathari N. Gichuhi, Max Petzold, Elizabeth Sullivan, Frank Taulo, Özge Tunçalp, Rachel Vanderkruik

**Affiliations:** ^1^ Department of Obstetrics and Gynecology University of Campinas São Paulo Brazil; ^2^ UNDP–UNFPA–UNICEF–WHO–World Bank Special Programme of Research Development and Research Training in Human Reproduction (HRP) Department of Reproductive Health and Research WHO Geneva Switzerland; ^3^ Department of Infectious Disease Epidemiology London School of Hygiene and Tropical Medicine London UK; ^4^ Department of Obstetrics and Gynecology University of Utah Salt Lake City UT USA; ^5^ Department of Medicine Warren Alpert School of Medicine Brown University Providence RI USA; ^6^ Molecular and Clinical Sciences Research Institute St George's University of London UK

**Keywords:** Cohort studies, Disability, Functionality, Pregnancy, Pregnancy complications, Severe maternal morbidity, Validation studies

## Abstract

**Objective:**

To validate the WHO Disability Assessment Schedule 2.0 (WHODAS 2.0) 12‐item tool against the 36‐item version for measuring functioning and disability associated with pregnancy and the occurrence of maternal morbidity.

**Methods:**

This is a secondary analysis of the Brazilian retrospective cohort study on long‐term repercussions of severe maternal morbidity (SMM) among women who delivered at a tertiary facility (COMMAG study). We compared WHODAS‐12 and WHODAS‐36 scores of women with and without SMM using measures of central tendency and variability, tests for instruments’ agreement (Bland‐Altman plot), confirmatory factor analysis (CFA), and Cronbach alpha coefficient for internal consistency.

**Results:**

The COMMAG study enrolled 638 women up to 5 years postpartum. Although the median WHODAS‐36 and ‐12 scores for all women were statistically different (13.04 and 11.76, respectively; *P*<0.001), there was a strong linear correlation between them. Furthermore, the mean difference and the differences in variance analyses demonstrated agreement of total scores between the two versions. CFA demonstrated how the WHODAS‐12 questions are divided into six previously defined factors and Cronbach alpha showed good internal consistency.

**Conclusion:**

WHODAS‐12 demonstrated agreement with WHODAS‐36 for total score and was a good instrument for screening functioning and disability among postpartum women, with and without SMM.

## Abstract 2

WHODAS‐12 demonstrated agreement with WHODAS‐36 and was good for screening functioning and disability among women who experienced pregnancy with or without severe maternal morbidity.

## 1 INTRODUCTION

According to the United Nations World Report on Disability, more than 1 billion people in the world live with some form of disability, of which nearly 200 million experience considerable difficulties in functioning. Globally, people with disabilities have poorer health outcomes, lower educational achievements, less economic participation, and higher rates of poverty than people without disabilities.^1^ However, the burden of ill health associated with pregnancy‐related and obstetric complications is yet to be completely understood because of the broad impact of short‐ and long‐term consequences.^2^ Functioning and disability among women of reproductive age is poorly studied. The use of a simple and effective tool to identify and measure disability in the postpartum period is key to improving maternal health worldwide.^2^, ^3^

The WHO has made efforts to address the problem of identifying and assessing disability and functioning by establishing an international classification system, the International Classification of Functioning, Disability and Health (ICF).^4^ All standard instruments for measuring disability and health needed to be linked conceptually and operationally to the ICF to allow comparisons across different cultures and populations using these new concepts. Using the ICF's conceptualization of disability, WHO developed a new tool—the WHO Disability Assessment Schedule (WHODAS)—to measure difficulties in performing daily activities in a more simplified manner. Like ICF, the tool was designed to “assess the limitations on activity and restrictions on participation experienced by an individual, irrespective of medical diagnosis”.^5^ The WHODAS tool was refined to include cross‐cultural measurement of health status and to respond to calls for improving the scope and cultural adaptability of the original WHODAS. Its second version (WHODAS 2.0) was presented as a general measure of functioning impairment and disability in major life domains. The WHODAS 2.0 instrument intends to measure activity function and participation in daily activities in the 30 days preceding its application.^5^, ^6^ The instrument has three versions, two of which were compared in this analysis. The complete 36‐question version (WHODAS‐36) was administered, and the results of the abbreviated 12‐question version (WHODAS‐12) were compared with those of the full version simply by extracting and analyzing the relevant subset of 12 questions. The possibility of using a shorter version of the instrument, the WHODAS‐12, is appealing when planning population screening surveys; however, WHODAS‐12 has neither been tested nor validated among pregnant women. For each domain of the original WHODAS‐36, the 12‐item version includes two sentinel items with good screening properties that identify over 90% of individuals with mild functioning impairment, based on all 36 items, in general populations.^5^

WHODAS 2.0 was translated into, culturally adapted to, and validated for various languages, including Brazilian Portuguese, for a study that implemented it among postpartum women with and without severe maternal morbidity (SMM).^7^, ^8^ This retrospective cohort study included 638 women who delivered at a tertiary public hospital in Brazil. Women with SMM showed increased WHODAS‐36 scores (functioning impairment) compared with women without SMM.^9^

The objectives of the current analysis were to compare and validate the abbreviated WHODAS‐12, using the complete WHODAS‐36 as the reference, for assessing postpartum disability among women (both with and without maternal morbidity) who delivered up to 5 years before assessment.

## 2 MATERIALS AND METHODS

This is a secondary analysis of the Brazilian retrospective cohort study, known as COMMAG, on the long‐term repercussions of SMM on women who delivered at a tertiary maternity unit (between July 2008 and June 2012).^9^ The methods have previously been published.^9^, ^10^ Briefly, WHODAS 2.0 was applied to a cohort of women with and without the diagnosis of SMM (potentially life‐threatening conditions and maternal near‐miss incidents), according to WHO standard definition and criteria.^11^ Score calculations for the analysis used the WHODAS “item‐response‐theory” (IRT) based scoring.^5^

After obtaining individual informed consent, face‐to‐face interviews were carried out by healthcare professionals specially trained for the study. All women meeting the SMM criteria who delivered during the study period were invited to participate and a control group (1:1 rate) was also selected. For each woman who experienced SMM (“exposed” group), a woman without SMM (“nonexposed group”), irrespective of other less‐severe morbidities, and who delivered the same year and at the same institution, was recruited.

The full version of WHODAS 2.0 (36 items) has six domains. The first domain, “cognition” (six questions), evaluates communication and thinking activities, including concentration, memory, problem solving, learning, and communication. The second domain assesses “mobility” (five questions), including physical capabilities such as standing up, moving around inside the house, going outside the house, and walking a long distance. The third domain, “self‐care” (four questions), measures an individual's capacity to carry out needs such as hygiene, getting dressed, eating, and staying alone. The fourth domain, “relationship with people” (five questions), examines interactions with others, and difficulties that an individual with adverse health conditions may encounter. The fifth domain, on “life activities” (eight questions), evaluates difficulty with daily activities (household responsibilities, leisure, work, and school). The last domain, “participation” (eight questions), assesses social dimensions, such as engaging in community activities, barriers and obstacles to such interactions, and other problems, such as maintenance of personal dignity.^6^ Response options for every question are: no difficulty, little, moderate, severe, and extreme difficulty.

To study WHODAS‐12, the questions common to WHODAS‐36 were labelled from S1 to S12 (Table 1) and these comprise two corresponding questions from each WHODAS‐36 domain (sentinel key questions).^6^ It should be noted that if the woman does not work or study, the number of questions in the versions reduces to 11 and 32, respectively (this impacts the analysis discussion as some questions will have missing values). Both the full and short versions of WHODAS 2.0 generate an overall score ranging from 0 to 100 (0=no disability; 100=full disability).

**Table 1:** WHODAS‐12 and WHODAS‐36 questions.^a^

WHODAS‐12	WHODAS‐36
S1	D 2.1
S2	D 5.1
S3	D 1.4
S4	D 6.1
S5	D 6.5
S6	D 1.1
S7	D 2.5
S8	D 3.1
S9	D 3.2
S10	D 4.1
S11	D 4.2
S12	D 5.5

a Complete and translated tools can be accessed at: http://www.who.int/classifications/icf/form_whodas_downloads/en/.

To perform the comparison between WHODAS‐36 and WHODAS‐12 scores, we analyzed the overall median, mean, and standard deviation. Differences in medians were tested using the nonparametric Wilcoxon signed rank test because of skewed distributions. In addition to the measures of central tendency, the Pearson correlation coefficient was used to address linear correlation between WHODAS‐36 and WHODAS‐12 total scores through the different dispersion values. Then, to evaluate the mean and variance of the difference between WHODAS‐12 and WHODAS‐36 scores, we used the Bland‐Altman plot with Pitman test of difference in variance. Both methods were used to evaluate agreement between the short and full versions of WHODAS 2.0.^12^, ^13^ These analyses were repeated to compare each domain across the two tools. The score for each domain was proportionally converted to a score also ranging from 0 to 100.

Additionally, we compared the 5th, 10th, 25th, 50th, 75th, 90th, and 95th percentiles (with their respective 95% confidence intervals) of total score for both groups (with and without morbidity), using McNemar test to evaluate statistical difference between WHODAS‐12 and WHODAS‐36 through the different cut‐off points for each percentile. A *P* value of 0.05 or below was considered statistically significant. Finally, we performed a confirmatory factor analysis (CFA) and Cronbach α to evaluate the intercorrelation between WHODAS‐12 questions and domains, and to measure the internal consistency of the short instrument, respectively. CFA was applied according to the Kaiser‐Meyer‐Olkin (KMO) measure of sampling adequacy, and the method used for extraction was the Varimax method with Kaiser Normalization.

The study protocol was assessed and approved by the local Institutional Review Board of the School of Medical Sciences at the University of Campinas and complied with all ethical requirements for studies involving human beings in Brazil. Each woman was fully informed and signed an individual consent form before being enrolled. Participating women were initially approached and interviewed by phone using the computer assisted telephone interview (CATI) unit, at which time they were invited to the hospital for a visit. During this visit, additional evaluations were performed, including an assessment of the corresponding child.

## 3 RESULTS

The COMMAG study enrolled 638 women, 323 without and 315 with severe maternal morbidity. Their general characteristics are published elsewhere.^9^ In total, 631 women completed the entire WHODAS‐36 instrument, which in turn contains all the WHODAS‐12 questions. Missing information in any domain limits the calculation of the total scores and therefore seven women were excluded from the analysis. The results presented compare the 36‐item and 12‐item scores for all 631 women with complete data; the differences between scores in women with morbidity or without morbidity are explained in further detail elsewhere in this Supplement.^14^

**Figure 1:**
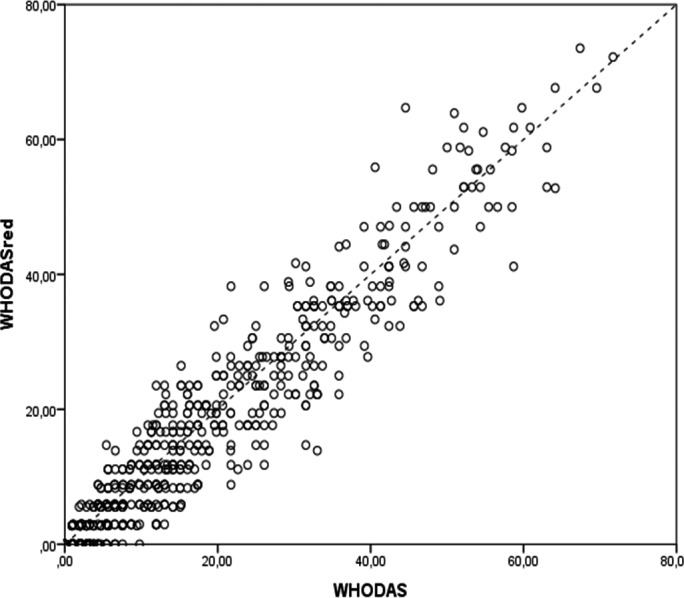
Correlation between the full and short version scores of the WHODAS 2.0 (n=631).

**Figure 2:**
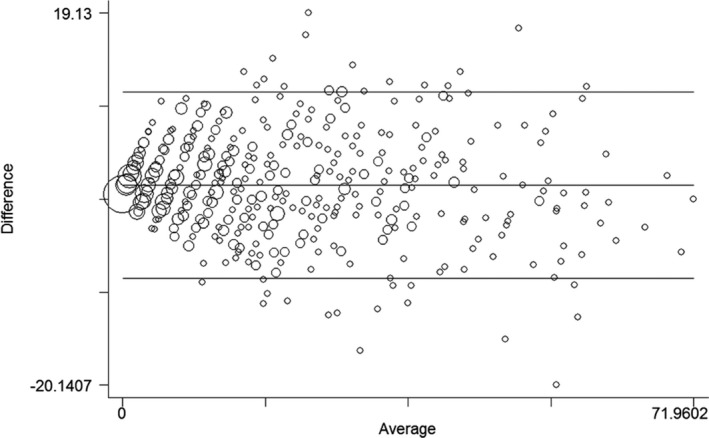
Bland‐Altman comparison of the 36‐ and the 12‐item versions of the WHODAS 2.0. Limits of agreement (reference range for difference): −8.866 to 10.766; mean difference: 0.950 (CI, 0.567–1.334); range: 0.000–71.960; Pitman test of difference in variance: *r*=−0.073, *P*=0.068, n=631.

In comparing the two instruments, the median of WHODAS‐36 and WHODAS‐12 total scores as well as those for each domain were assessed. The values for the total scores for the 631 women were 13.04 and 11.76 (*P*<0.001), respectively (Table 2). The domain scores demonstrate that only domain 4 showed no significant differences between medians (*P*=0.870). Although central tendency analyses were statistically different between the two versions, Figure 1 shows a very high linear correlation between WHODAS‐36 and WHODAS‐12 total scores (correlation coefficient of 0.945) and Figure 2 shows that the differences in variance of different scores were not statistically significant (*P*=0.068). Thus, the symmetrical distribution of the Bland‐Altman plot shows that WHODAS‐12 neither over‐ nor underestimates the total 36‐item version score.

**Table 2:** Mean and median scores of the full and short versions of the WHODAS 2.0 instrument for each domain and total score (n=638)

Domain	Score WHODAS‐36	Score WHODAS‐12	*P* value^a^
Domain 1			<0.001
Mean	21.4	17.5	
SD	18.2	19.8	
Median	15.0	12.5	
Domain 2			<0.001
Mean	13.8	18.0	
SD	19.0	23.5	
Median	6.3	12.5	
Domain 3^b^			<0.001
Mean	5.4	3.9	
SD	12.3	13.4	
Median	0.0	0.0	
Domain 4			0.870
Mean	14.6	14.3	
SD	20.2	23.5	
Median	8.3	0.0	
Domain 5^b^			0.029
Mean	22.2	23.5	
SD	26.9	30.2	
Median	10.0	0.0	
Domain 6			<0.001
Mean	20.4	23.7	
SD	20.7	28.1	
Median	12.5	16.7	
Total score^b^			<0.001
Mean	17.4	16.4	
SD	15.6	16.0	
Median	13.0	11.8	

Abbreviation: SD, standard deviation.

a Related samples Wilcoxon signed rank test.

b Missing information.

**Figure 3:**
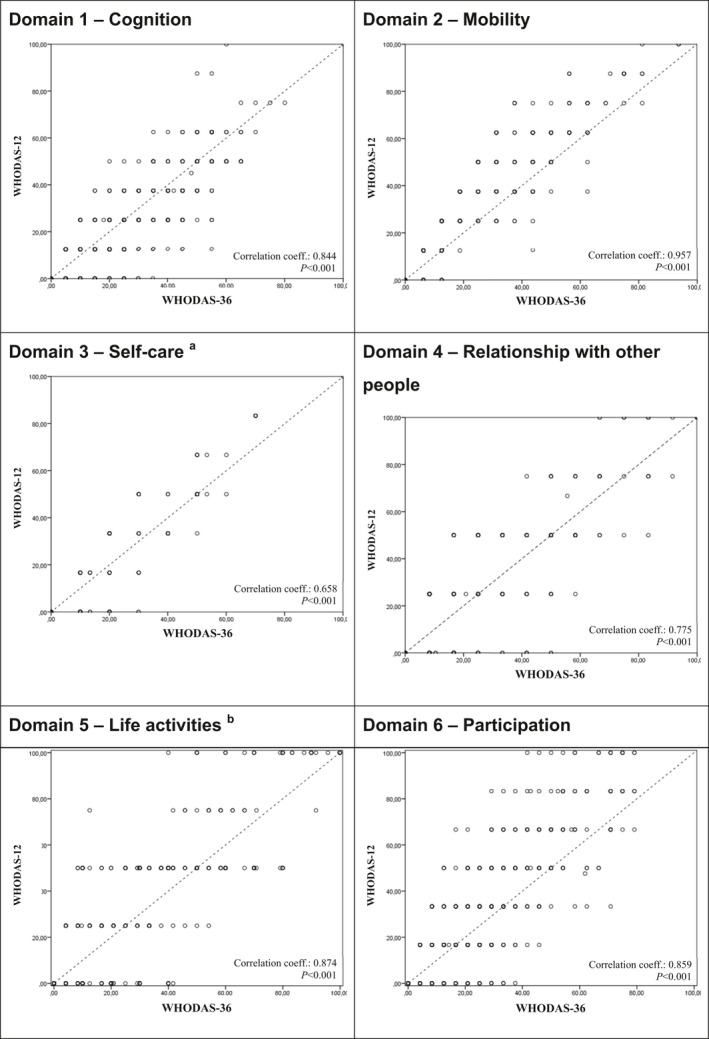
Correlation between the full and short version scores for each domain of the WHODAS 2.0 (n=638). Abscissa: Domain of WHODAS‐36; Ordinate: Domain of WHODAS‐12; Missing data (number of women) for: *a*=1, *b*=6.

**Figure 4:**
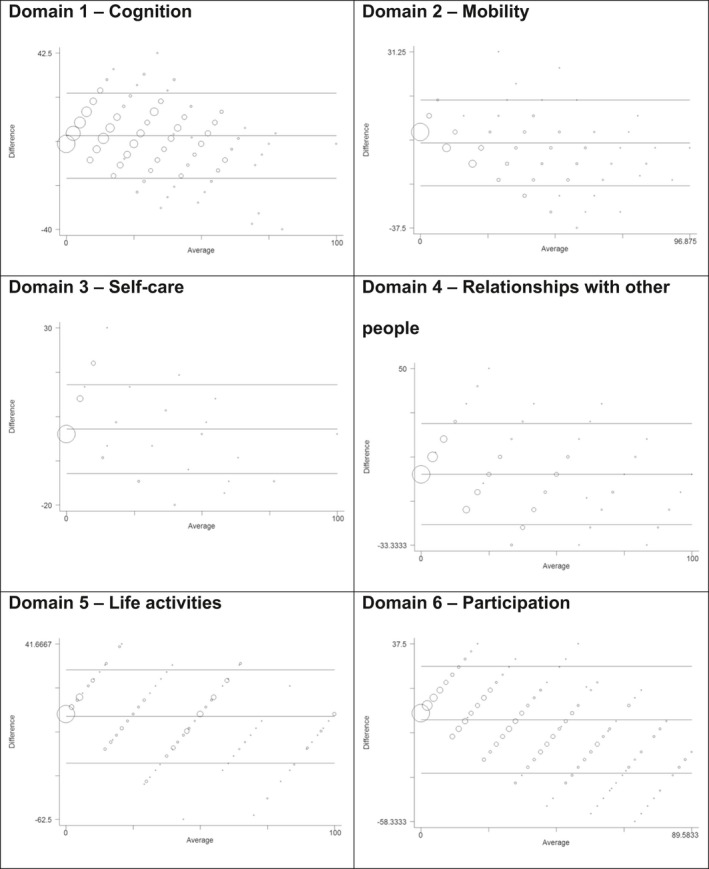
Bland‐Altman comparison of the 36‐ and the 12‐item versions for each domain of the WHODAS 2.0 (n=638). *Domain 1*: Limits of agreement (reference range for difference): −16.078 to 23.870; mean difference: 3.896 (CI, 3.119–4.672); range: 0.000–100.000; Pitman test of difference in variance: *r*=−0.161, *P*<0.001 (n=638). *Domain 2*: Limits of agreement (reference range for difference): −20.932 to 12.414; mean difference: −4.259 (CI, −4.907 to −3.611); range: 0.000–96.875; Pitman test of difference in variance: *r*=−0.547, *P*<0.001 (n=638). *Domain 3*: Limits of agreement (reference range for difference): −11.054 to 13.974; mean difference: 1.460 (CI, 0.973–1.947); range: 0.000–100.000; Pitman test of difference in variance: *r*=−0.168, *P*<0.001 (n=637). *Domain 4*: Limits of agreement (reference range for difference): −23.667 to 24.129; mean difference: 0.231 (CI −0.698 to 1.160); range: 0.000–100.000; Pitman test of difference in variance: *r*=−0.288, *P*<0.001 (n=638). *Domain 5*: Limits of agreement (reference range for difference): −28.995 to 26.392; mean difference: −1.302 (CI, −2.383 to −0.220); range: 0.000–100.000; Pitman test of difference in variance: *r*=−0.243, *P*<0.001 (n=632). *Domain 6*: Limits of agreement (reference range for difference): −32.166 to 25.527; mean difference: −3.320 (CI, −4.441 to −2.198); range: 0.000–89.583; Pitman test of difference in variance: *r*=−0.533, *P*<0.001 (n=638).

In general, there was a high linear correlation between total scores of WHODAS‐36 and WHODAS‐12, with correlation coefficients ranging from 0.658 (domain 3) to 0.957 (domain 2) (Fig. 3). However, the analyses of mean differences and difference in variance by domains were different from those observed for the total score, showing significant variance between domain scores of the two tool versions (Fig. 4). The mean difference ranged from 0.231 (domain 4) to −4.259 (domain 2).

**Figure 5:**
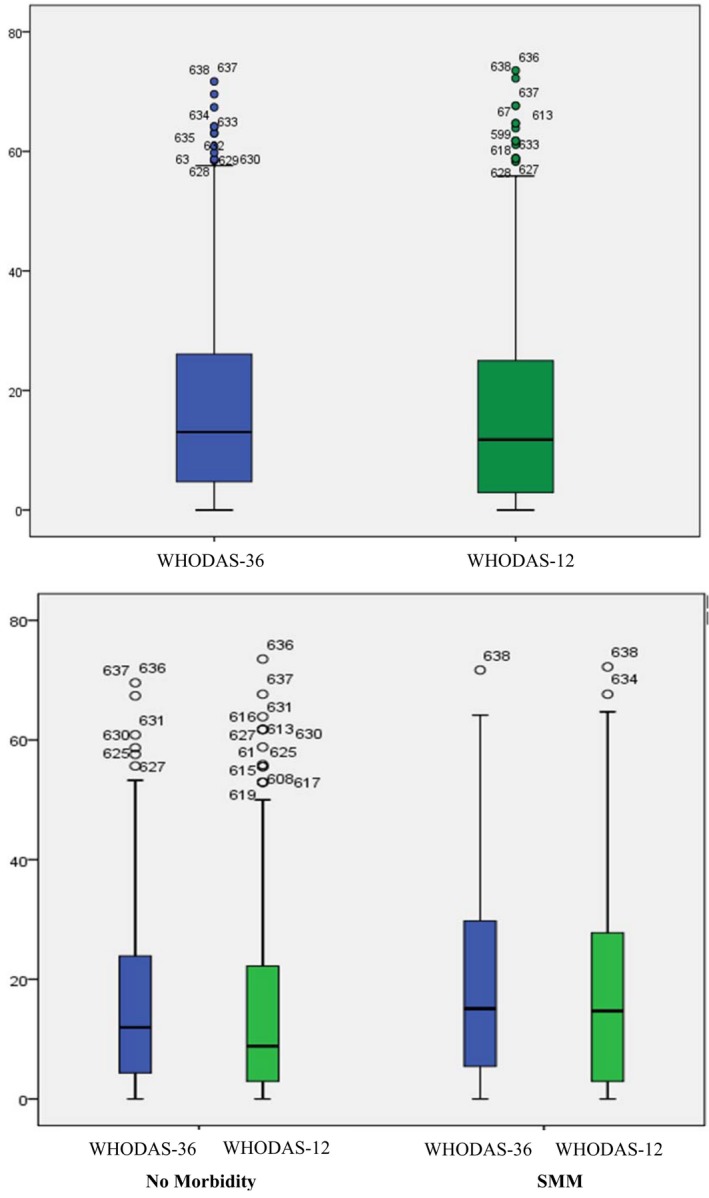
Boxplot of total scores of WHODAS‐12 and WHODAS‐36 for all women in the COMMAG study (n=631). [Colour figure can be viewed at http://www.wileyonlinelibrary.com]

Table 3 shows percentiles of the WHODAS‐12 and WHODAS‐36 total scores for all women and by history of SMM. The WHODAS‐12 percentile scores were similar to those of the WHODAS‐36 for the total sample (all women regardless of SMM status) in all percentiles. Figure 5 shows the percentile scores of the total sample, and a breakdown of scores by women's history of SMM using a boxplot. Although there was a statistically significant difference (*P*=0.003) when comparing the 36‐ and 12‐item scores in the 25th percentile for women with SMM and the 50th percentile for women without SMM, we considered these differences very small and not clinically relevant.

**Table 3:** Score values of different percentiles of the full and short versions of the WHODAS 2.0 instrument for women with severe maternal morbidity (n=311) and women without morbidity (n=320)

Percentile and groups	WHODAS‐36	WHODAS‐12	*P* value^a^
	Score (95% CI)	Score (95% CI)	
Percentile 5			
No morbidity	0.9 (0.0–1.1)	0.0 (0.0–0.0)	
SMM	0.0 (0.0–0.9)	0.0 (0.0–0.0)	
Total	0.0 (0.0–0.9)	0.0 (0.0–0.0)	
Percentile 10			
No morbidity	1.9 (1.1–1.9)	0.0 (0.0–0.0)	
SMM	1.0 (0.0–2.0)	0.0 (0.0–0.0)	
Total	1.1 (0.9–1.9)	0.0 (0.0–0.0)	
Percentile 25			
No morbidity	4.4 (3.3–5.4)	2.9 (2.8–5.6)	0.701
SMM	5.4 (3.8–7.1)	2.9 (2.8–5.6)	0.003
Total	4.7 (3.8–5.4)	2.9 (2.8–5.6)	0.243
Percentile 50			
No morbidity	12.0 (9.4–13.0)	8.8 (8.3–11.8)^b^	0.014
SMM	15.1 (13.0–17.1)	14.7 (11.8–17.7)^b^	0.572
Total	13.0 (12.0–14.2)	11.8 (11.1–13.9)	0.082
Percentile 75			
No morbidity	23.9 (20.7–27.3)	22.2 (19.4–25.0)	0.839
SMM	30.2 (26.1–33.0)	27.8 (25.0–32.4)	>0.999
Total	26.1 (24.53–29.3)	25.0 (23.5–27.8)	0.451
Percentile 90			
No morbidity	36.8 (32.6–42.4)	36.0 (32.4–41.5)	>0.999
SMM	44.6 (39.6–48.3)	41.2 (38.2–50.0)	0.227
Total	41.3 (37.0–44.5)	38.2 (36.1–43.3)	0.076
Percentile 95			
No morbidity	47.0 (41.6–52.2)	47.2 (41.2–55.6)	>0.999
SMM	53.9 (46.7–58.5)	51.1 (47.1–58.3)	>0.999
Total	50.4 (45.7–53.8)	50.0 (46.5–55.6)	0.146

Abbreviations: SMM, severe maternal morbidity; CI, confidence interval.

a Dependent samples, McNemar Test, cut‐off point: each percentile; percentiles 5 and 10 were not compared.

b *P*=0.009 (Independent samples Mann‐Whitney test).

The CFA with six factors showed that both questions from domains 1, 2, 3, and 5 are grouped in factors 4, 2, 1, and 3, respectively (Table 4). Questions from domains 4 and 6 were not grouped with the corresponding question of the same domain. These factors could explain 64.3% of variance in CFA. Cronbach α for the 12 questions (n=297) and for the 6 domains (n=631) was 0.840 and 0.794, respectively. The analysis for the 11‐item version (n=631), excluding question D55a (work/school activities) showed similar reliability (Cronbach α=0.815).

**Table 4:** Confirmatory factor analysis (CFA) of the Brazilian Portuguese version of the WHODAS 2.0 questionnaire in 12 questions and 6 factors (Generalized Least Squares method).^a^

Question and its main topic	Factor^b^^,^ c					
	1	2	3	4	5	6
D11a Concentration	0.137	0.109	0.195	**0.766**	0.061	0.163
D14a Learning a new task	0.025	0.118	0.144	**0.458**	0.125	0.037
D21a Standing for long periods	0.227	**0.449**	0.193	0.210	0.019	0.100
D25a Walking a long distance	0.179	**0.938**	0.200	0.123	0.093	0.151
D31a Washing your whole body	**0.870**	0.184	0.141	0.112	0.024	0.093
D32a Getting dressed	**0.695**	0.140	0.179	0.061	0.104	0.046
D41a Dealing with strangers	0.129	0.134	**0.397**	0.165	0.276	0.072
D42a Maintaining a friendship	0.080	0.058	0.238	0.171	**0.946**	0.087
D51a Household responsibilities	0.233	0.345	**0.458**	0.247	0.207	0.160
D55a Work/school activities	0.207	0.203	**0.660**	0.248	0.084	0.259
D61a Joining in community activities	0.216	0.205	**0.523**	0.294	0.279	0.209
D65a Health affects one's emotions	0.125	0.231	0.325	0.213	0.119	**0.874**
Variance, %	12.71	11.98	11.89	9.85	9.61	8.21
Cumulative variance, %	12.70	24.69	36.58	46.44	56.06	64.26

a The Kaiser‐Meyer‐Olkin (KMO) measure of sampling adequacy suggested that the sample is factorable (KMO=0.866, *P*<0.001; Extraction method: Maximum likelihood).

b Rotated method: Varimax method with Kaiser Normalization.

c The highest factor value for each question is given in bold.

## 4 DISCUSSION

Our validation study indicated that WHODAS‐12 is a good substitute for WHODAS‐36. We found a very high correlation between the total scores of WHODAS‐12 and WHODAS‐36, but relatively poor agreement at the sublevel of specific domains. Reassuringly, the agreement between the versions did not seem to vary significantly according to different levels of functionality (different percentile values). Finally, the confirmatory factor analyses validated the internal consistency and reliability of WHODAS‐12.

The use of WHODAS‐36 in 631 women (315 women who experienced SMM and 323 who did not) (COMMAG study)^9^ has provided us with a great opportunity to assess the abbreviated 12‐item version, enabling the validation of a potentially reproducible, manageable, short, and reliable instrument to assess women's functionality. The utilization of standardized instruments is key for evaluating and comparing the long‐term consequences of SMM across studies, sites, and complication diagnoses. This complex challenge is essential for advancing the promotion of maternal health, particularly in the context of the Sustainable Development Goals, which place emphasis on the whole woman and her ability to work and participate in economic and social activities.^2^, ^3^, ^9^, ^10^, ^15^ These efforts follow WHO's standardization of the concepts of and criteria for “maternal near miss” and “potentially life‐threatening” in 2009,^11^ which led to calls for similar standardization efforts for less‐severe maternal morbidities and their consequences during and after pregnancy.^3^, ^16^

The 12‐item version of WHODAS 2.0 has a similar structure to WHODAS‐36, and comprises two questions for each domain, one with low and the other with high complexity regarding the functionality (domain) that is being assessed.^5^ When applied to a general population, the short version has shown high correlation and agreement with WHODAS‐36, yet it requires significantly less time for its implementation (about 5 minutes)—a clear potential advantage of the shorter version.^5^ The statistically significant difference between the median WHODAS‐12 and WHODAS‐36 scores is to be expected considering the relatively large sample size of the COMMAG study. Minor differences are more likely to be statistically significant in large studies, although, in this case, it seems clinically irrelevant, given the overall low scores presented across the entire group of 631 postpartum women.

The fact that some questions did not group as expected in the CFA may be a result of the development of the WHODAS‐36. Originally, it was meant to identify self‐perception of multidimensional disabilities using the total score or individual scores for each of the six domains.^6^ Questions in domains 4 and 6 were not grouped with the same factor in the CFA, as was expected (and as we found) with questions in the other domains. Questions D61a and D65a were originally part of a different matrix of the ICF instrument, addressing different aspects of functionality such as community, social and civic life (Question D61a), and mental function (Question D65a), which could explain the differences in the CFA grouping for these questions.

According to our analyses, even when considering the WHODAS total score percentiles for women with or without SMM, the WHODAS‐12 total score seemed to agree with that of the full version. Though our analysis focused on comparing the 36‐ and 12‐item scores across all women, regardless of morbidity status, the subanalysis was undertaken to ensure that there would be no differences regardless of the broad range of values presented. The only two statistically significant differences between WHODAS‐12 and WHODAS‐36 percentiles were found in the 25th and 50th percentiles, for SMM and no morbidity groups, respectively, with low overall scores, most likely with no clinical significance. However, future studies should address the issue of percentile cut‐off points for WHODAS score among postpartum women and its association with impaired functioning.

Additionally, the mean difference of Bland‐Altman analyses of the total score demonstrated that the short version may not under‐ or overestimate the score generated by WHODAS‐36. In contrast, the scores of each WHODAS‐12 domain showed good correlation but not agreement with those of WHODAS‐36, as previously described.^5^, ^6^ Therefore, although the short version might not identify specific disabilities, it showed equivalent performance in evaluating functional impairment in the total WHODAS score, and could be recommended for that purpose in future studies.

WHODAS‐12 is a short version of a more refined and robust instrument, composed of selected questions from the WHODAS‐36, thus, a high correlation between the two versions is not surprising. The Bland‐Altman analysis is a useful method to address the agreement between the versions by constructing limits of agreement calculated using the mean and the standard deviation of the two scores^17^ and has been used before to validate quality of life and functioning instruments.^18^, ^19^ A study of correlation, instead of calculating the variance of the average scores (study of the differences), is occasionally performed to address agreement between different instruments, but it can be considered a misleading approach for this purpose.^12^

Another study, performed by Üstun et al.,^5^ compared WHODAS‐12 with other instruments that also assess functional impairment—including FIM (Functional Independent Measure), LHS (London Handicap Scale), SF‐12 and SF‐36 (Short Form Health Surveys), and WHOQOL (WHO Quality of Life)—and showed that the 12‐item version of WHODAS 2.0 has good concurrent validity for general populations. Additionally, when comparing the values for the total scores on the 36‐item version (13.04) and 12‐item version (11.76) for the 631 women, these scores place the COMMAG study population above the 72nd population percentile of the general adult population.^5^ These findings suggest there may be an effect of pregnancy on functioning in the postpartum period, though more research is necessary to confirm this.

Although the COMMAG study included a large number of women, there are some limitations regarding the methods and findings. The interval between pregnancy and the evaluation of functioning using WHODAS 2.0 ranged from 1 to 5 years. The different intervals of assessment may have biased the demonstration of an association between the occurrence of SMM and disabilities, although previous analyses did not show such impact.^9^ Additionally, although WHODAS‐36 and WHODAS‐12 are shown to be valuable in assessing functional impairment and disability in women who experienced uncomplicated pregnancies or SMM, there is still no clear cut‐off point to discriminate between women with or without disability. A recent systematic review of the consequences of maternal morbidity on health‐related functioning found more than 130 articles published between 2005 and 2014 meeting the inclusion criteria. However, the review concluded that the evaluation of functioning domains was generally poor, highlighting the importance of developing a specific instrument for pregnant and postpartum women.^20^

Additional secondary analyses and further studies aiming to validate a reliable cut‐off for this population would be valuable. The recent recognition of the importance and advances in the conceptualization of maternal morbidity for maternal health care highlights the need for surveillance of the repercussions of maternal complications. Future studies need to address the identification of domains specific to women during pregnancy and the postpartum period (including important functioning for the pregnant or postpartum circumstances, e.g. ability to breastfeed, care, and response to the needs of newborns) that could be added to the instrument. This in turn would clarify the burden of pregnancy itself and SMM, enabling early identification of adverse outcomes and the implementation of intervention packages and strategies to prevent and treat them.^2^, ^3^, ^21^

The total scores derived from the short version of WHODAS showed good agreement with scores on the 36‐item version, and it was found to be a good instrument for screening women with pregnancy‐associated functional impairment and disability. The use of a simple, short, and reproducible tool to measure disability in the postpartum period is crucial to improving maternal health worldwide, especially considering the adverse repercussions of severe maternal morbidity. A cut‐off for both versions would be a step forward in evaluating functionality and remains a great challenge to be addressed. Future research focusing on functional impairment may improve maternal health and enable awareness of issues not frequently considered during postpartum care.

## AUTHOR CONTRIBUTIONS

JGC, RCP, MAP, MLC, and CS first developed the idea and planned the study. JGC, RCP, MAP, MLC, CS, and ECF implemented and conducted the study. CS, RTS, ECF, JM, JPG, MLC, and JGC planned the current ancillary analysis and led the manuscript writing process. MHS conducted the data analysis. VF, KB, TF, PvD, DC, and MB provided inputs for the current analysis and participated in the editing process, providing essential direction and advice for finalizing the manuscript. LS conceptualized the maternal morbidity measurement initiative. All authors read and approved the final manuscript.

## ADDITIONAL COMMAG AND MMWG GROUP MEMBERS

**COMMAG members:** Carla B. Andreucci, Carina R. Angelini, Juliana P. Ferraz, Dulce M. Zanardi, Rodrigo S. Camargo. **MMWG members:** Sara Cottler, Olubukola Fawole, Luis Gadama, Atf Ghérissi, Gill Gyte, Michelle Hindin, Anoma Jayathilaka, Amanda Kalamar, Yacouba Kone, Nenad Kostanjsek, Isabelle Lange, Laura A. Magee, Arvind Mathur, Affette McCaw‐Binns, Mark Morgan, Stephen Munjanja, Gathari N. Gichuhi, Max Petzold, Elizabeth Sullivan, Frank Taulo, Özge Tunçalp, Rachel Vanderkruik.

## CONFLICTS OF INTEREST

The authors declare that they have no conflicts of interest.
